# Fusion with and without lever reduction in degenerative lumbar spondylolisthesis: a retrospective study

**DOI:** 10.1186/s13018-023-04507-9

**Published:** 2024-01-03

**Authors:** Chao Kong, Dongfan Wang, Wei Wang, Yu Wang, Shibao Lu

**Affiliations:** 1 Department of Orthopedics, Xuanwu Hospital, Capital Medical University, No. 45 Changchun Street, Xicheng District, Beijing, China; 2 National Center for Clinical Research on Geriatric Diseases, No. 45 Changchun Street, Xicheng District, Beijing, China

**Keywords:** Lumbar spine, Degenerative lumbar spondylolisthesis, Lever reduction, In situ fusion, Clinical outcomes, Radiological assessments, Complications

## Abstract

**Background:**

The reduction of slipped vertebra is often performed during surgery for degenerative lumbar spondylolisthesis (DLS). This approach, while potentially improving clinical and radiological outcomes, also carries a risk of increased complications due to the reduction process. To address this, we introduced an innovative lever reduction technique for DLS treatment. This study aims to investigate the clinical efficacy, radiological outcomes, and complications of fusion with or without lever reduction.

**Methods:**

We conducted a retrospective review of prospectively collected data from a registry of patients who underwent lumbar fusion surgery for DLS, with a follow-up of at least 24 months. Self-reported measures included visual analog scale (VAS) for back or leg pain, Oswestry Disability Index (ODI), and the achievement of minimal clinically important difference (MCID). Radiological assessments encompassed spondylolisthesis percentage (SP), focal lordosis (FL), and lumbar lordosis (LL). Complications were categorized using the modified Clavien–Dindo classification (MCDC) scheme. Patients were assigned to the reduction group (RG) and non-reduction group (NRG) based on the application of the lever reduction technique. Clinical and radiological outcomes at baseline, immediately after surgery, and at the last follow-up were compared.

**Results:**

A total of 281 patients were analyzed (123 NRG, 158 RG). Baseline patient demographics, comorbidities, and surgical characteristics were similarly distributed between groups except for operating time (NRG 129.25 min, RG 138.04 min, *P* = .009). Both groups exhibited significant clinical improvement after surgery (all, *P* = .000), with no substantial difference between groups (VAS, ODI, or the ability to reach MCID). Patients in RG showed statistically lower SP and higher FL during follow-up (all, *P* = .000). LL was comparable at different time points within each group or at the same time point between the two groups (all, *P* > .050). The overall complication rate (NRG 38.2%, RG 27.2%, *P* = .050) or specific complication rates per MCDC were similar between groups (all, *P* > .050). Patients in RG were predisposed to a lower risk of adjacent segment degeneration (ASDeg) (NRG 9.8%, RG 6.3%, *P* = .035).

**Conclusions:**

There were no significant differences in postoperative measures such as VAS scores for back and leg pain, ODI, the ability to reach MCID, overall complication rate, or specific complication rates per MCDC between surgical approaches. However, fusion with lever reduction demonstrated a notable advantage in restoring segmental spinal sagittal alignment and reducing the occurrence of ASDeg compared to in situ fusion.

## Background

Degenerative lumbar spondylolisthesis (DLS) is a common pathological condition in the elderly population, characterized by the anterior displacement of a superior vertebra over the adjacent caudal vertebra, while the neural arch remains intact [1, 2]. Due to the spinal canal stenosis, compression of the nerve root in the lateral recess or in the foramen, and segmental instability secondary to spondylolisthesis, patients with DLS usually present with neurogenic claudication, radicular leg pain, or back pain [3]. In severe cases or when conservative treatments fail, decompression of the affected neural structures and stabilization of the spinal segment, so-called decompression and fusion surgery, are considered as a means to provide satisfactory long-term results [4, 5]. Besides the aforementioned interventions, whether or not to reduce the spondylolisthesis intraoperatively still needs to be determined by surgeons. Theoretically, the reduction procedure contributes to reducing slip distance, increasing segmental lumbar lordosis or intervertebral disc height, and potentially leads to better clinical outcomes or a higher fusion rate [6–9]. Nonetheless, conventional reduction methods predominantly rely on distraction of the disc space and direct elevating pull of the pedicle screws, which may also introduce a higher risk of complications, such as neurologic deficits, hardware failure (screw loosening or pull out), prolonged operating time, or loss of reduction [6, 10–13].

To reduce the surgical-related complications linked to the reduction procedure, we introduced a composite reduction technique encompassing both traditional elevating-pull reduction and innovative lever reduction. The clinical utility of this technique was previously demonstrated in case series [14]. This study aims to delve deeper into the clinical efficacy, radiological outcomes, and complications associated with fusion with or without lever reduction for DLS treatment. By doing so, we intend to offer valuable insights into the comparative effectiveness and safety of these two surgical approaches for DLS.

## Methods

### Patient population

Following approval by the ethics committee at our hospital, a retrospective review of the spine registry data was conducted on a consecutive cohort of 488 patients diagnosed with lumbar spondylolisthesis between May 2015 and December 2020. All the clinical and radiological data had been prospectively collected at the respective follow-up visits. Inclusion criteria were as follows: (1) single-level DLS (Meyerding grade I or II), (2) refractory to conservative treatments for more than 6 months, and (3) at least 2 years’ of follow-up with complete clinical and radiological data. Patients with other types of spondylolisthesis, multilevel (≥ 2) spondylolisthesis, high-grade spondylolisthesis (Meyerding grade III or IV), hip disorders, previous spinal surgery or trauma, or incomplete data, were excluded from analysis.

### Surgical techniques

**Fig. 1:**
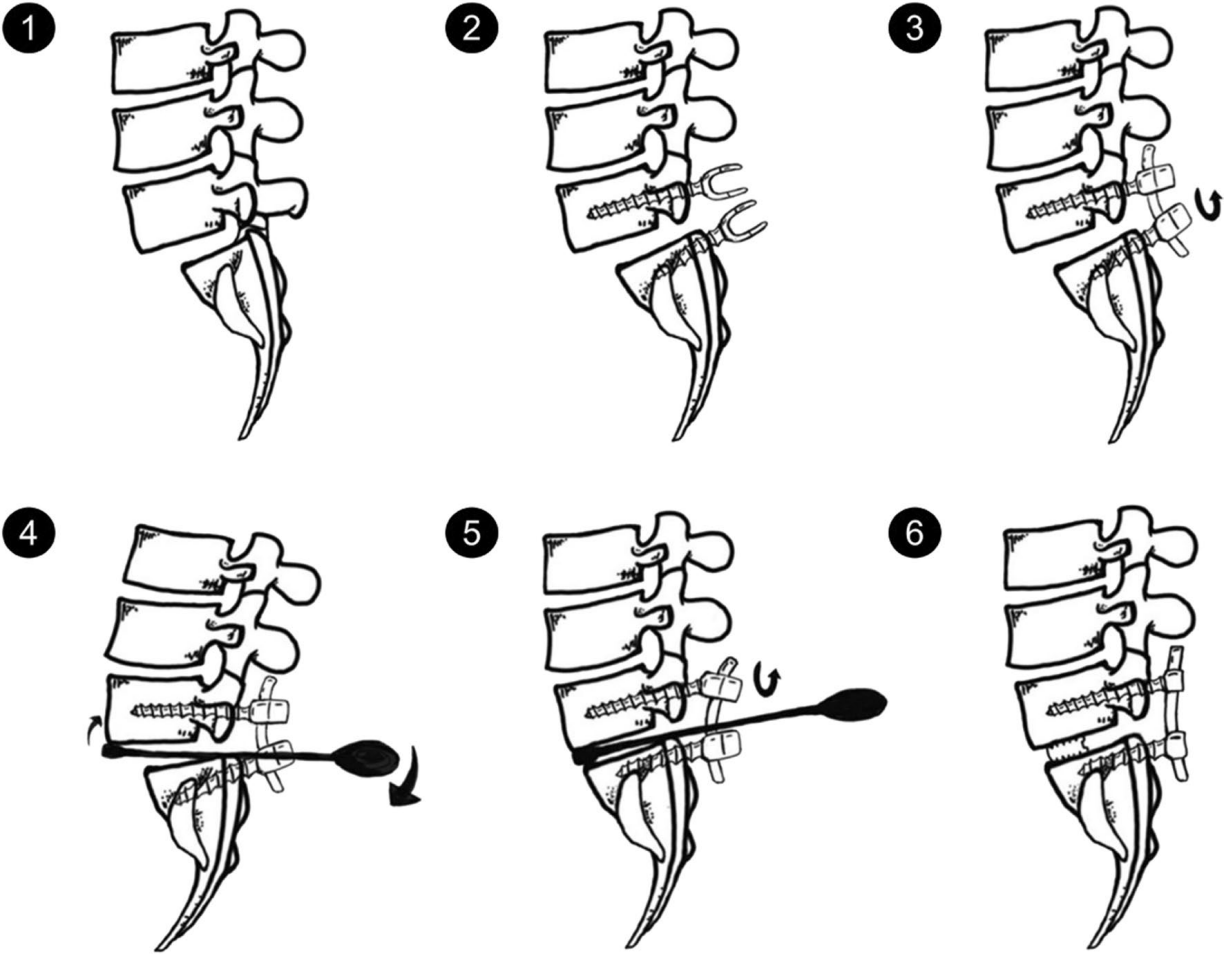
Reduction process of a slipped vertebrae. (1) Forward slippage of L5; (2) pedicle screws were placed at both vertebra of the slipped levels; (3) the nerve roots were decompressed before reduction. After removal of the disk tissues and endplate preparation, a rod was placed unilaterally and the pedicle screw of the lower vertebrae was locked; (4) a lever repositioner was placed at the anterior rim of the slipped vertebrae under fluoroscopy; (5) with the lower vertebrae as the lever fulcrum, force was applied to gradually pry the slipped vertebrae upward; 6) the pedicle screws of the slipped vertebrae were locked. Then, an addition rod was placed and all screws were locked. Quote from the study by Chao et al. (https://doi.org/10.1186/s12891-019-3028-8)

All included patients experienced stenosis caused by DLS and underwent decompression and lumbar interbody fusion during subsequent surgery. The surgeries were performed through an open posterior midline approach. Before bony decompression, bilateral pedicle screws were placed. Decompression consisted of bilateral facetectomy and partial foraminotomy, including the hypertrophic ligament flavum. The disc space was opened and thoroughly cleaned with intradiscal drills and pituitary rongeurs. The cartilaginous endplates were cleaned with caution so as to not cause injury to the bone endplates. Bilateral nerve roots were liberated before reduction. The reduction of the slipped vertebra was conducted following lever reduction technique (Fig. 1) [14]. The extent of slip reduction was verified with fluoroscopy. Then, the interspace was packed with autologous bone graft material, and an appropriately sized polyetheretherketone cage filled with bone was inserted into the disc space.

Patients undergoing fusion with lever reduction were assigned to the reduction group (RG). Conversely, patients undergoing in situ fusion (where intentional surgical reduction was not performed) were assigned to the non-reduction group (NRG). The assignment was made per surgeon's choice.

### Clinical measurements

Clinical assessments including visual analog scale (VAS) for back pain, VAS for leg pain, and Oswestry Disability Index (ODI). The VAS was utilized to measure the severity of back and leg pain for patients based on a 10-cm line, with “painless” (0) and “most severe pain” (10 cm) at each respective end [15]. The validated ODI is a self-administered questionnaire for evaluating back-specific functional disability, consisting of 10 items with scores from 0 to 5, and higher ODI indicates more severe disability [16]. Minimal clinically important difference (MCID) was introduced to analyze the clinical significance of variations in clinical outcomes [17]. MCID values were set at 14.9 points for ODI, 2.1 points for VAS back pain, and 2.8 points for VAS leg pain [18]. All clinical outcomes were assessed by research assistants before surgery, immediately after surgery, and at each follow-up.

### Radiological data acquisition

**Fig. 2:**
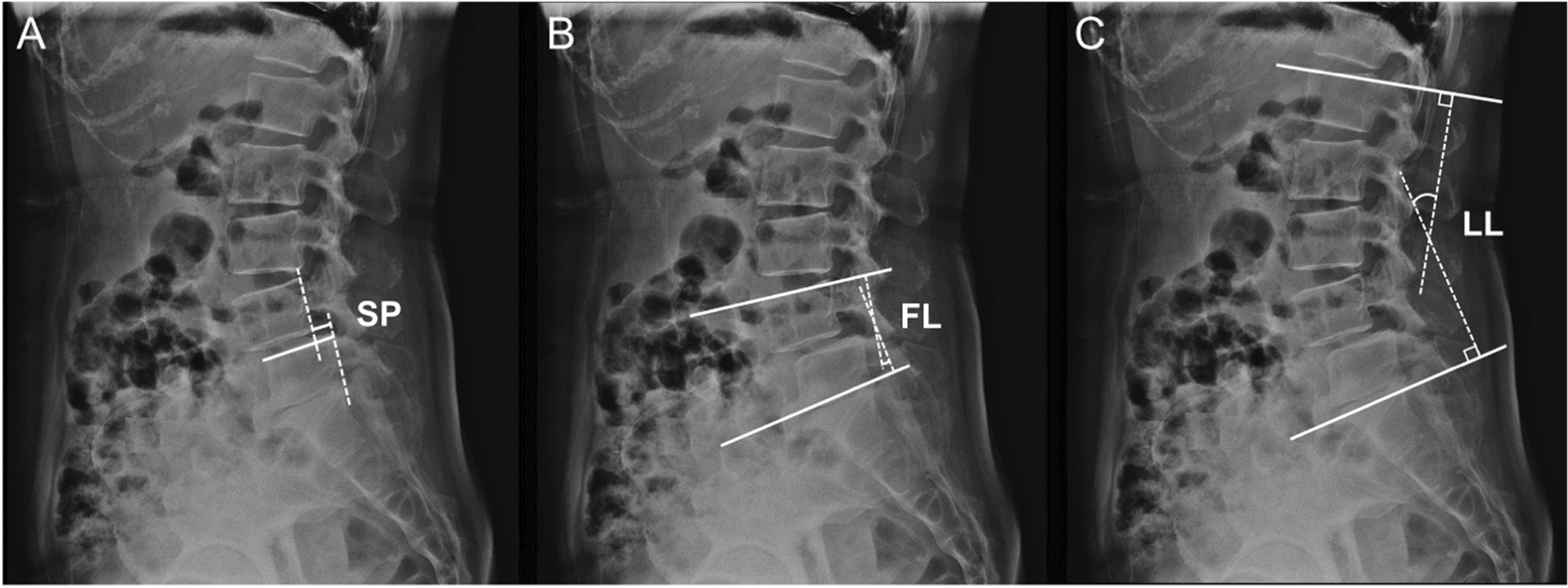
Illustration of the radiological measurements. **A** SP, spondylolisthesis percentage, the ratio of the interval between two extended lines of the posterior aspect of superior slipped vertebra and the inferior normal vertebra to the length of the superior endplate of the inferior normal vertebra; **B** FL, focal lordosis, the Cobb angle between the superior endplate of the upper slipped vertebra and the inferior endplate of the lower normal vertebra; **C** LL, lumbar lordosis, the Cobb angle between the superior endplates of both L1 and S1

Measurements of radiological parameters are illustrated in Fig. 2, covering: (1) spondylolisthesis percentage (SP), the ratio of the interval between two extended lines of the posterior aspect of superior slipped vertebra and the inferior normal vertebra to the length of the superior endplate of the inferior normal vertebra; (2) focal lordosis (FL), the Cobb angle between the superior endplate of the upper slipped vertebra and the inferior endplate of the lower normal vertebra; and (3) lumbar lordosis (LL), the Cobb angle between the superior endplates of both L1 and S1. All radiological measurements were taken by two trained spinal surgeons (WW and YW) before surgery, immediately after surgery, and at each follow-up. The average of two measurements was taken as the final result.

### Complications assessment

All complications were recorded in light of the modified Clavien–Dindo classification (MCDC) scheme containing five types of complications: Type I–normal recovery without any treatment; Type II–pharmacologic treatment needed; Type III–invasive intervention under general anesthesia needed; Type IV–intensive care unit admission needed; Type V–death [19]. Adjacent segment degeneration (ASDeg) was diagnosed when plain radiographs, computerized tomography, or magnetic resonance imaging demonstrated one or more of the following lesions at the segment adjacent to the fused segment that were not present preoperatively: (1) development of anterolisthesis or retrolisthesis > 4 mm, (2) range of motion between adjacent vertebral bodies > 10°, (3) loss of disc height > 10%, (4) osteophyte formation > 3 mm, as well as (5) spinal stenosis caused by facet joint hypertrophy, compression fracture, or degenerative scoliosis [20, 21]. Symptomatic ASDeg requiring reoperation was diagnosed as adjacent segment disease (ASDis). Radiographic fusion was assessed using Bridwell's grading criteria, and both grades I and II were considered radiographic signs of successful fusion, while grades III and IV vice versa [22]. Pedicle screw or cage loosening was defined as a radiolucency of ≥ 1 mm around the screw or the cage [23].

### Statistical analysis

Data were analyzed using SPSS Statistics software (version 26.0, IBM Corp., Armonk, NY, USA). Statistical significance was set at a level of *P* < 0.05.

Continuous data are reported as mean values ± standard deviation. The assumption of normal distribution for the data was verified using the Shapiro–Wilk test. The independent samples t test and the Mann–Whitney U test were employed for intergroup comparison in each time point. The paired t test was used for the intra-group comparison of different time points. The Chi-square test was utilized to compare categorical variables between groups. Intraclass correlation coefficients (ICC) were calculated to evaluate the inter-rater reliability of radiographic assessments. ICC values below 0.5, between 0.5 and 0.75, between 0.75 and 0.9, and above 0.90 indicate poor, moderate, good, and excellent reliability, respectively.

## Results

### Demographics

**Fig. 3:**
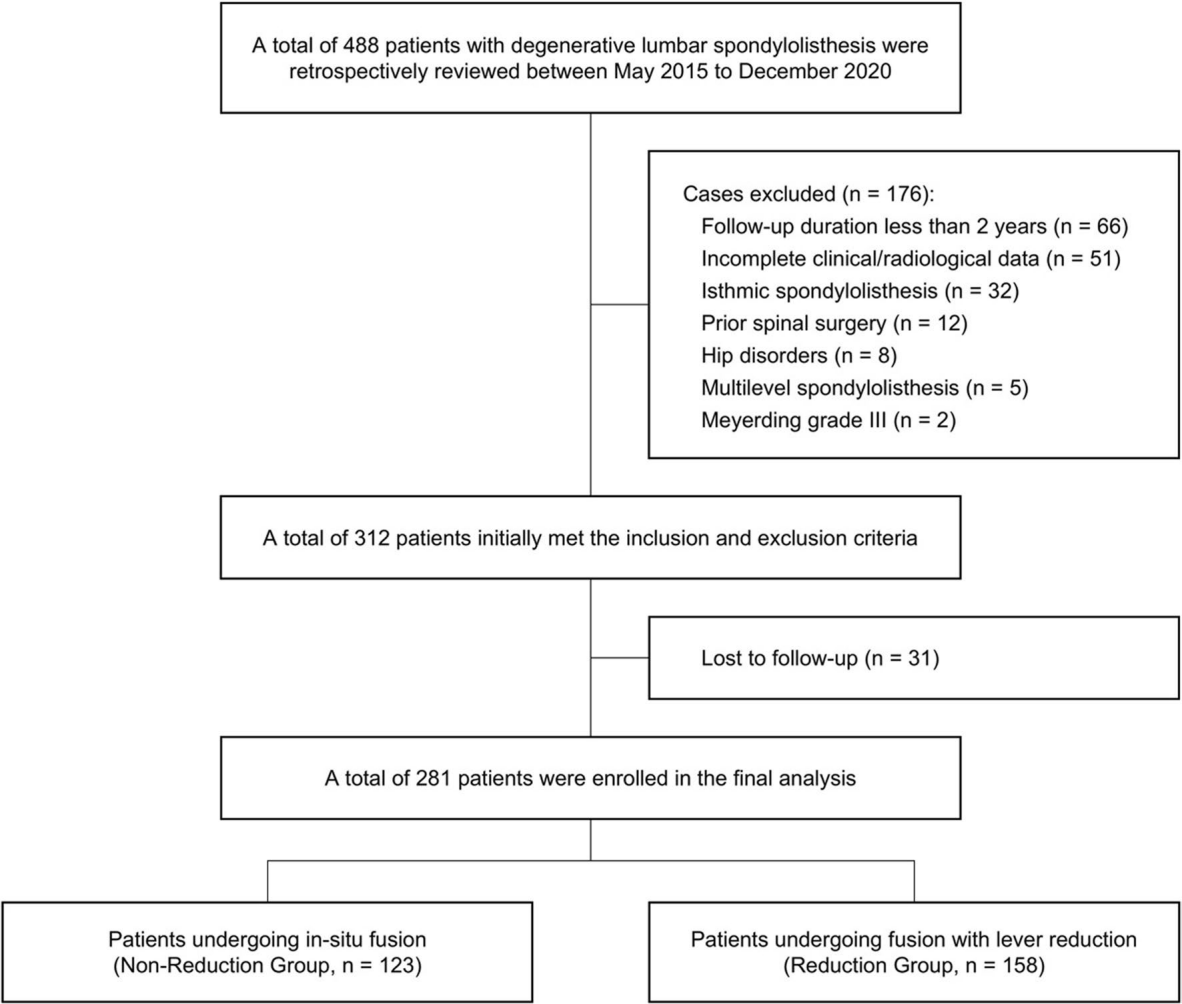
Screening procedure of the patients

A total of 312 patients initially met the inclusion and exclusion criteria. However, 31 patients (9.94%) were lost to follow-up. Among the remaining 281 patients, 123 underwent in situ fusion, while 158 underwent fusion with lever reduction (Fig. 3). The enrolled patients were followed up for an average duration of 29 months, ranging from 24 to 41 months. The gender distribution was similar in both groups, with the majority being female (NRG: 74.8% vs. RG: 70.9%, *P* = 0.466). Surgery at the L4-L5 level was the most common for both groups (NRG: 77.2% vs. RG: 70.9%, *P* = 0.231). Notably, there was a significant difference in operating time between the groups (NRG: 129.25 ± 27.41 min vs. RG: 138.04 ± 28.02 min, *P* = 0.009), while no statistically significant differences were observed in other demographic metrics (Table 1).

**Table 1:** Demographic data of the patients

Variables	NRG (*n* = 123)	RG (*n* = 158)	*P*
Age (year)	71.81 ± 7.03	72.32 ± 7.71	.612
Gender (female/male)	92/31	112/46	.466
Currently smoker	19	23	.836
Alcohol use	30	47	.318
Body mass index (kg/m^2^)	25.65 ± 3.83	24.72 ± 4.09	.055
Comorbidities			
Hypertension	32	52	.210
Diabetes	24	35	.590
Coronary artery disease	7	16	.178
Anxiety	3	2	.777
Depression	1	1	1.000
Osteoporosis	18	29	.407
Meyerding grade (I/II)	72/51	87/71	.560
Surgical level			
L3–L4	16	29	.137
L4–L5	95	112	.231
L5–S1	12	17	.784
Operating time (mins)	129.25 ± 27.41	138.04 ± 28.02	.009**
Blood loss (ml)	238.37 ± 112.9	251.46 ± 132.68	.383
Length of hospital stay (day)	10.04 ± 1.94	9.74 ± 2.10	.221

**P < 0.01

### Patient-reported outcomes

In terms of back pain measured by the VAS, there was a remarkable reduction in NRG from 4.89 ± 1.45 preoperatively to 1.80 ± 1.32 postoperatively (*P* = 0.000) and 1.95 ± 1.12 at the final follow-up. Similarly, in RG, VAS back pain significantly improved from 5.06 ± 1.43 before surgery to 1.73 ± 1.21 (*P* = 0.000) postoperatively and 1.81 ± 1.14 at the last follow-up. However, no substantial differences in back pain intensity were observed between the two groups at corresponding evaluation time points. Following surgery, 70.7% of NRG patients and 78.5% of RG patients achieved MCID, though the statistical difference was not significant (*P* = 0.136) (Table 2).

**Table 2:** Clinical measures of the patients

Variables	NRG (*n* = 123)	RG (*n* = 158)	*P*
Visual analog scale for back pain			
Preoperation	4.89 ± 1.45	5.06 ± 1.43	.307
Postoperation	1.80 ± 1.32†	1.73 ± 1.21†	.680
Follow-up	1.95 ± 1.12	1.81 ± 1.14	.315
Reached MCID (*n* [%])	87 (70.7%)	124 (78.5%)	.136
Visual analog scale for leg pain			
Preoperation	5.00 ± 1.82	5.17 ± 1.74	.444
Postoperation	1.84 ± 1.45†	1.78 ± 1.20†	.739
Follow-up	1.56 ± 1.10¶	1.73 ± 1.16	.211
Reached MCID (*n* [%])	96 (78.0%)	130 (82.3%)	.375
Oswestry Disability Index			
Preoperation	49.40 ± 11.58	48.61 ± 10.45	.552
Postoperation	15.99 ± 11.08†	16.35 ± 9.07†	.763
Follow-up	15.37 ± 8.99	16.87 ± 6.28	.099
Reached MCID (*n* [%])	98 (79.7%)	127 (80.4%)	.883

*MCID* minimal clinically important difference

^†^The discrepancy was statistically different between preoperative and postoperative values

^¶^The discrepancy was statistically different between postoperative and last follow-up values

When considering VAS leg pain, there was a decrease from 5.00 ± 1.82 to 1.84 ± 1.45 postoperatively (*P* = 0.000) and 1.56 ± 1.10 at the last follow-up in NRG. In RG, VAS leg pain decreased from 5.17 ± 1.74 before surgery to 1.78 ± 1.20 (*P* = 0.000) postoperatively and 1.73 ± 1.16 at the last follow-up. Comparable VAS leg pain scores were noted between groups at corresponding assessment time points. The proportion of patients achieving MCID was similar between NRG (78.0%) and RG (82.3%) without a statistically significant difference (*P* = 0.375) (Table 2).

A similar decreasing trend was evident in the ODI, with scores reducing from 49.40 ± 11.58 to 15.99 ± 11.08 postoperatively (*P* = 0.000) and 15.37 ± 8.99 at the last follow-up in NRG. In RG, preoperative ODI decreased from 48.61 ± 10.45 to 16.35 ± 9.07 postoperatively (*P* = 0.000) and 16.87 ± 6.28 at the last follow-up. No statistically significant differences were detected between groups at any assessment time points. The proportion of patients achieving MCID was similar between NRG (79.7%) and RG (80.4%), with no significant statistical difference (*P* = 0.883) (Table 2).

### Radiological outcomes

Results of ICC analysis indicated good or excellent reliability for all radiographic assessments (SP: 0.771, FL: 0.816, LL: 0.901).

The preoperative SP was 21.68% ± 6.18% in NRG and 20.25% ± 6.46% in RG. This value significantly decreased to 16.85% ± 6.23% (*P* = 0.000) and 5.69% ± 4.31% (*P* = 0.000), respectively. At the final follow-up, SP increased to 19.70% ± 7.67% (*P* = 0.000) in NRG and 7.67% ± 4.43% (*P* = 0.000) in RG. Notably, patients who underwent fusion with lever reduction consistently exhibited significantly lower SP during the follow-up period compared to those who underwent in situ fusion (Table 3).

**Table 3:** Radiological outcomes of the patients

	Spondylolisthesis percentage (%)			Focal lordosis (°)			Lumbar lordosis (°)		
	Preoperation	Postoperation	Follow-up	Preoperation	Postoperation	Follow-up	Preoperation	Postoperation	Follow-up
NRG	21.68 ± 6.18	16.85 ± 6.23†	19.70 ± 7.67¶	11.58 ± 6.10	13.99 ± 6.22†	13.85 ± 6.39	41.01 ± 10.19	41.61 ± 10.17	42.22 ± 10.60
RG	20.25 ± 6.46	5.69 ± 4.31†	7.67 ± 4.43¶	12.68 ± 6.01	17.13 ± 5.90†	17.07 ± 5.98	41.93 ± 11.18	42.26 ± 11.17	41.78 ± 11.29
*P*	.062	.000**	.000**	.130	.000**	.000**	.479	.614	.739

**P < 0.01

^†^The discrepancy was statistically different between preoperative and postoperative values

^¶^The discrepancy was statistically different between postoperative and last follow-up values

The preoperative FL was similar between the two groups (NRG: 11.58° ± 6.10° vs. RG: 12.68° ± 6.01°, *P* = 0.130). However, FL significantly increased to 13.99° ± 6.22° (*P* = 0.000) in NRG and 17.13° ± 5.90° (*P* = 0.000) in RG after surgery. There were no statistically significant differences in FL at the last follow-up compared to the postoperative values in both groups. Patients in the RG demonstrated greater FL both postoperatively and at the last follow-up compared to those in the NRG (Table 3).

Regarding LL, no statistically significant differences were observed at different time points within each group or at the same time point between the two groups (Table 3).

### Complications and reoperations

A total of 90 complications were documented based on the MCDC classification, comprising 47 complication (Type I: 28, Type II: 16, Type III: 3) in the NRG and 43 complications (Type I: 24, Type II: 17, Type III: 2) in the RG, respectively. Patients undergoing in situ fusion demonstrated a higher incidence of ASDeg compared to those undergoing fusion with lever reduction (NRG: 9.8% vs. RG: 6.3%, *P* = 0.035). L4-5 reduction would be more beneficial in terms of prevention of ASDeg; however, a larger sample size is still needed to validate this finding. No significant differences were observed between the groups in the proportion of ASDis, cage malposition, cerebrospinal fluid leakage, unsuccessful fusion, residual pain or numbness, screw loosening, wound infection, and other general complications (Table 4).

**Table 4:** Frequency and type of complications

	NRG (*n* = 123)	RG (*n* = 158)	*P*
Total complications (*n* [%])	47/123 (38.2%)	43/158 (27.2%)	.050
MCDC Type I complications (*n* [%])	28/123 (22.8%)	24/158 (15.2%)	.105
MCDC Type II complications (*n* [%])	16/123 (13.0%)	17/158 (10.8%)	.561
MCDC Type III complications (*n* [%])	3/123 (2.4%)	2/158 (1.3%)	.777
Details of complications (*n* [%])			
Adjacent segment degeneration (ASDeg)	17/123 (9.8%)	10/158 (6.3%)	.035*
ASDeg at L2–L3	1/16 (6.3%)	0	.356
ASDeg at L3–L4	13/95 (13.7%)	8/112 (7.1%)	.120
ASDeg at L4–L5	3/12 (25.0%)	2/17 (11.8%)	.645
Adjacent segment disease (ASDis)	1/123 (0.8%)	1/158 (0.6%)	1.000
Cage malposition	1/123 (0.8%)	0	.438
Cerebrospinal fluid leakage	2/123 (1.6%)	3/158 (1.9%)	1.000
Non-union of the fused level	4/123 (3.3%)	2/158 (1.3%)	.467
Residual pain	3/123 (2.4%)	2/158 (1.3%)	.777
Residual numbness	1/123 (0.8%)	2/158 (1.3%)	1.000
Screw loosening	2/123 (1.6%)	3/158 (1.9%)	1.000
Wound infection	1/123 (0.8%)	1/158 (0.6%)	1.000
Constipation	6/123 (4.9%)	4/158 (2.5%)	.458
Diarrhea	1/123 (0.8%)	0	.438
Delirium	1/123 (0.8%)	0	.438
Lower-limb thrombosis	1/123 (0.8%)	2/158 (1.3%)	1.000
Nausea and vomiting	5/123 (4.1%)	9/158 (5.7%)	.543
Pneumonia	0	1/158 (0.6%)	1.000
Urinary retention	1/123 (0.8%)	2/158 (1.3%)	1.000
Urinary tract infection	0	1/158 (0.6%)	1.000
Causes of reoperation (n [%])			
ASDis	1/123 (0.8%)	1/158 (0.6%)	1.000
Cage malposition	1/123 (0.8%)	0	.438
Wound infection	1/123 (0.8%)	1/158 (0.6%)	1.000

*P < 0.05

Two patients, one from each group, required revision surgery due to ASDis. One patient in the NRG experienced recurrent pain caused by cage malposition 3 months postoperatively and resolved through reoperation. Wound infection was observed in two patients, with one case identified in each group, necessitating postoperative debridement.

## Discussion

The necessity of a concomitant reduction procedure during the fusion surgery for DLS remains a controversial topic. Currently, conventional wisdom suggests that the reduction of spondylolisthesis holds theoretical appeal due to its potential for indirect decompression of neuroforamina and restoration of the sagittal lumbosacral alignment. Within this context, multiple reduction approaches, such translation reduction, distract and slip reduction, cantilever technique, or minimally invasive slip reduction, have been developed and utilized in the treatment of DLS [24–27]. However, the implementation of these methods relies upon adequate contact force between the instrumentation and vertebra and might instead result in implant-related complications, especially for elderly DLS patients with diminished bone quality. In response to these challenges, our clinical center introduced a novel lever reduction procedure in combination with transforaminal lumbar interbody fusion [14]. The present study compared the clinical efficacy, radiographic outcomes, and complications of in situ fusion versus fusion with lever reduction in a cohort of 281 patients. Results of our study highlighted the benefits associated with lever reduction in terms of restoring segmental sagittal alignment and reducing complications, while no superiority of the additional reduction procedure over in situ fusion in improving clinical outcomes was exhibited.

The impact of reduction on clinical outcomes in lumbar spondylolisthesis remains uncertain, as comparative studies have yielded conflicting results. A randomized trial conducted by Lian et al. involving 73 patients with DLS revealed similar postoperative VAS, ODI, and Japanese Orthopedic Association (JOA) scores between patients who underwent fusion with or without reduction [6]. Another study involving 65 patients with symptomatic spondylolisthesis, conducted by Heo et al., demonstrated that intraoperative reduction led to greater improvements in ODI after surgery [8]. Conversely, Tay et al. did not find any significant clinical benefits associated with reduction in cohorts with low-grade spondylolisthesis [23]. Regarding high-grade spondylolisthesis, a recent meta-analysis indicated that slip reduction correlated with more substantial overall enhancements in ODI when compared to in situ fusion [28]. In our current investigation, we did not identify a connection between spondylolisthesis reduction and improvements in clinical outcomes or an increased proportion of patients achieving the MCID (Table 2). Considering that the majority of patients in this study showed only slight degenerative spondylolisthesis, one plausible explanation for this result might be that the indirect decompression effect resulting from reduction was marginal when contrasted with the direct decompression achieved during the fusion procedure. Therefore, the reduction procedure had minimal effect on clinical improvement.

In line with previous findings, our results also indicate that the focal lordosis increases significantly as the spondylolisthesis percentage decreases [6, 8]. In contrast, the overall lumbar lordosis shows variation with no substantial differences across the three assessment time points, whether reduction was performed or not (Table 3). From a practical standpoint, establishing a connection between the restoration of spinal alignment and the perceived enhancements in treatment effectiveness for patients is crucial. In a prospective study enrolling 57 patients with DLS who underwent lumbar fusion surgery, Kuhta et al. reported that obtaining adequate SL was correlated with favorable ODI 5 years postoperatively [29]. Similarly, Takahashi et al. showed that DLS patients with a higher increase in SL were predisposed to a higher JOA recovery rate after lumbar fusion surgery for DLS [30]. On the contrary, loss of overall lumbar lordosis resulted in a higher risk of poor clinical outcomes [31]. Therefore, despite our inability to identify a statistical distinction in clinical outcomes between NRG and RG as mentioned earlier, the significance of the reduction procedure remains worthy of contemplation since it both improves segmental morphology and maintains overall lordosis, which provides potential therapeutic benefit in patients with DLS.

The choice of the most appropriate surgical plan for a surgeon can be influenced by the complications associated with various surgical techniques. However, there remains a lack of consensus regarding the definition and grading of complications arising from spine surgeries. In this study, all complications were categorized according to the MCDC system [19]. The results indicated that patients undergoing fusion with lever reduction were inclined to experience a lower overall complication rate and MCDC Type I complication rate compared to those undergoing in situ fusion, although this difference was not statistically significant. Regarding specific categories, the reduction technique exhibited a distinct advantage in reducing the incidence of ASDeg compared to in situ fusion (Table 4). This difference might be attributed to the increased FL resulting from the reduction procedure. As reported in previous studies, the proper restoration of FL curbs the compensatory increase in mobility and loading at the adjacent fused segment, thereby delaying the degeneration process [32, 33]. Moreover, the additional stresses during the reduction maneuvers might induce a higher risk of screw loosening or even pullout, as previously reported [6, 34]. Nonetheless, such negative effects were not evident in patients who underwent fusion with the lever reduction procedure in our research (Table 4). The lever device's distractive force can mitigate the pull force exerted on the instrumentation to some extent, potentially leading to fewer implant-related complications [14]. Considering these factors collectively, the superiority of fusion with lever reduction is primarily manifested in reducing the risk of complications rather than enhancing patient-reported outcomes. We believe that fusion with lever reduction could emerge as a viable alternative for DLS patients and is worthy of application, contributing to an enhanced long-term prognosis.

Our study has certain limitations that need to be acknowledged. Firstly, the retrospective nature of our study made it challenging to completely eliminate selection bias and attrition bias. Secondly, the decision to pursue lever reduction was primarily influenced by surgeon preferences and, in some cases, the availability of the lever reduction device. This introduces the possibility of unmeasured factors affecting the decision-making process, not accounted for in our study. Thirdly, only patients undergoing fusion with lever reduction and in situ fusion were included in the analysis. Therefore, the present study cannot conclusively prove the superiority or inferiority of lever reduction technique compared to other reduction techniques. The ongoing data collection of relevant research may address this gap in the future. Lastly, it is important to note that our cohort size was relatively small, which might marginally impact the robustness of our conclusions. Despite these limitations, our study yields valuable insights into the efficacy and safety of the innovative lever reduction technique for DLS. Furthermore, it contributes previously unavailable data that can help reconcile the ongoing debate surrounding fusion options with or without reduction.

## Conclusions

We conducted a comparison of clinical effectiveness, radiological outcomes, and complications between fusion with and without the innovative lever reduction technique in a group of 281 patients with DLS. There were no significant differences in postoperative measures such as VAS scores for back and leg pain, ODI, the ability to reach MCID, overall complication rate, or specific complication rates per MCDC between surgical approaches. However, a notable advantage was observed in fusion with lever reduction compared to in situ fusion in terms of restoring segmental spinal sagittal alignment and reducing the occurrence of ASDeg. In the long-term perspective, fusion with lever reduction might be a considerable alternative for the treatment of DLS.

## Data Availability

The datasets are used and/or analyzed during the current study available from the corresponding author on reasonable request.

## Abbreviations

DLS: Degenerative lumbar spondylolisthesis
RG: Reduction group
NRG: Non-reduction group
VAS: Visual analog scale
ODI: Oswestry Disability Index
MCID: Minimal clinically important difference
SP: Spondylolisthesis percentage
FL: Focal lordosis
LL: Lumbar lordosis
MCDC: Modified Clavien–Dindo classification
ASDeg: Adjacent segment degeneration
ASDis: Adjacent segment disease
JOA: Japanese Orthopedic Association
